# Nurses’ perception of the professional practice environment and its relation to organizational dehumanization and work passion

**DOI:** 10.1186/s12912-025-03241-3

**Published:** 2025-06-24

**Authors:** Shimaa Mohamed Salem, Huda Mohammed Bakeer, Hayam Ahmed El Shrief

**Affiliations:** Faculty of Nursing, Menoufia University, Menoufia, Egypt

**Keywords:** Organizational dehumanization, Nurses, Professional practice environment work passion

## Abstract

**Background:**

A professional practice environment enhances health worker recruitment and retention and contributes to quality patient care and health system strengthening. Hence, improving the professional practice environments of nurses at hospitals could improve the performance of the health system, increase work passion, and decrease organizational dehumanization.

**Aim:**

To assess nurses’ perceptions of the professional practice environment and its relation to organizational dehumanization and work passion.

**Method:**

A descriptive correlational research design was used. A convenience sample of staff nurses (n = 274) was recruited who worked in critical care units and inpatient departments at the National Liver Institute (NLI) in Shebin Elkom city/Menoufia Governorate, Egypt. Three instruments were used: the nursing professional practice environment questionnaire, the organization dehumanization scale, and the work passion scale.

**Results:**

More than two-thirds of the study subjects (72.3%) reported a favorable professional practice environment. The highest mean score and first ranking was related to the dimension of teamwork. The lowest mean score and lowest rank was associated with communication about patients. Additionally, more than two-thirds of the nurses studied perceived a feeling of not being dehumanized by their organization and a high level of passion toward their work (63% and 71.6%, respectively).

**Conclusion:**

There was a statistically significant negative correlation between the professional practice environment and organizational dehumanization. Moreover, there was a statistically significant positive correlation between the professional practice environment and work passion.

**Implication for nursing practice:**

Managers must design interventions to teach staff members that they are valued as individuals rather than expendable goods. To make staff members feel less dehumanized and more supported, hospitals and their managers may apply particular human resources practices, such as lowering workload, enhancing job stability, and providing training and development opportunities for their growth and grooming.

## Introduction

Enhancing the work environment of nurses during periods of staff scarcity is imperative, as the nursing workforce is one of the most critical components of the healthcare system for ensuring safe patient care. Consequently, practice environments may have a significant effect on healthcare organizations’ ability to deliver high-quality care to patients [1]. For the benefit of patients, nurses, and institutions, the professional practice environment is crucial. Additionally, professional practice settings enable nurses to work at the highest level of nursing practice, collaborate well with members of a multidisciplinary team, and rapidly mobilize resources [2].

At the same time, the environment in which healthcare professionals work can play a significant role in both attracting and keeping healthcare professionals. Additionally, elements of the setting for professional practice have an impact on nursing care quality both directly and indirectly [3]. The professional practice environment is referred to as “the organizational features of a workplace that either support or limit professional nursing practice” [4]. The World Health Organization (WHO) states that “an environment that attracts individuals into the health professions, motivates them to stay in the health workforce, and allows them to perform effectively can be characterized as an attractive and supportive work environment” [5].

A positive work environment (WE) in professional nursing practice environments supports safe practices and professional development. Key characteristics include quality-focused care, interdisciplinary cooperation, responsibility with authority, nursing leadership promotion, support for professional development, and cooperative relationships among health workers. These features benefit institutions, staff, and patients, reducing hospital mortality, readmissions, adverse events, and infection rates. Additionally, a positive WE is associated with the recruitment and retention of healthcare providers, which is critical during staffing shortages [2]. Because of this, negative work environments lead to job dissatisfaction, feelings of isolation, a desire to quit, and an increased risk of burnout, which negatively impacts the environment and patient outcomes [1].

A professional nursing practice is a work environment where policies, procedures, and systems are established to achieve organizational objectives and ensure job satisfaction. It allows nurses to meet personal needs, be productive, and provide excellent patient care. Benefits include improved organizational commitment, better retention of experienced nurses, higher-quality care, lower staff turnover, better patient outcomes, and increased nurse attraction [4].

The terms “work environment,” “working conditions,” and “job characteristics” are also used in scientific literature to refer to the professional practice environment. Nurse involvement, encouragement of supervisors, sufficient staffing, a patient-centered atmosphere, independence, a philosophy emphasizing the quality of care, cooperative relationships with peers and doctors, decentralization, and busyness are hallmarks of the professional work environment [6].

Nurse-organizational relationships are largely shaped by the professional work practice environment, which is a significant organizational feature. Nurses who work for organizations that offer professional supportive environments, such as cooperative interactions, learning opportunities, and emotional support, feel respected, trusted, and supported [7]. The establishment of a positive relationship between nurses and the organization increases the likelihood that their basic psychological needs will be met, which lessens the degree to which nurses feel dehumanized at work [8].

A concept that has recently gained attention as detrimental to both individuals and organizations is organizational dehumanization [9]. The term “organizational dehumanization” describes how nurses feel mistreated by their organization due to interactions in an unprofessional workplace, which frequently entails treating them like robots. Nurses do not treat people as human beings; rather, they treat them with less respect and use them as tools to achieve organizational objectives with less empathy and willingness. Nurses will perform worse and exhibit more impersonal attitudes. Additionally, stress and discontent with their jobs are more likely to be displayed by nurses who feel dehumanized by their supervisors [10].

Organizations may dehumanize themselves by mistreating their staff members. In these situations, staff members perceive themselves as interchangeable tools. Staff psychological health suffers as a result, and they become emotionally numb, stop thinking deeply, and become less empathetic. Also, staff resources are depleted, and negative perception impulses are suppressed by stressors [8].

When nurses feel that their psychological needs, such as competence, autonomy, and control, are not being satisfied, impressions of organizational dehumanization may arise. Organizational dehumanization is the denial or diminished attribution of humanity to managers or nurses, who may be perceived or experienced as inanimate objects. Higher levels of burnout are reported by nurses who believe that their supervisor is using them as a tool [11].

Additionally, the perception of dehumanization among nurses is reinforced by organizational inequity and intrinsic job design components such as repetition, activity fragmentation, and dependence [12]. Therefore, health care organizations that support professional nursing practices reduce nurses’ feelings of dehumanization by promoting autonomy, accountability, and control as well as by attracting and retaining nurses [9].

On the other hand, one important factor that may influence people’s job satisfaction and, in turn, their quality of work‒life balance is how passionate they are about their work. Passion is demonstrated by devoting time to one’s work, which affects performance [13]. When people are passionate about something, they genuinely devote much time and effort to it because they think it is crucial. As a result, a nurse’s passion for a certain activity greatly influences their life and can help to define who they are [14].

Passion is known as a strong preference for an activity that one finds enjoyable, feels necessary, and devotes a substantial amount of time and energy [15]. The primary feature of this work-related passion is its influence on professionals’ well-being, which can manifest either obsessive or harmonious passion, depending on the type. Harmonious passion is the type that permits resource allocation and significant investment in the workplace while preserving equilibrium with other aspects of life. Reduced burnout, life satisfaction, and job performance have all been linked to this harmonious passion. However, obsessive passion hinders a great balance between work and personal life by requiring a significant amount of time and effort [16].

Health teams and professionals are more stable and productive when they work with passion, which lowers employee turnover. Professional practice settings can foster a sense of passion at work. Nurse leaders can serve as role models for promoting passion at work by adding engaging tasks to inspire nurses and cultivate a culture of healthy competition, both of which increase the degree of work involvement and passion. Possibilities for personal growth and training can increase enthusiasm, passion, and job satisfaction [17].

## Significance of the study

The emphasis on quality care and adherence to evidence-based practice guidelines in the current healthcare environment has resulted in a new generation of knowledgeable and astute healthcare consumers. Consequently, nursing care in hospitals with professional practice environments is associated with lower hospital mortality, morbidity, length of stay, and care costs. A professional practice environment also influences patient care outcomes, enhances nursing care, and gives nurses more authority and control [18].

Additionally, dehumanizing experiences within organizations may be a prevalent problem in healthcare organizations at the moment. Therefore, there is a great need to shed light on this topic to plan preventive intervention programs for this phenomenon and offer hints about possible levers for practical reduction. A person’s degree of passion for work is also a crucial personal factor that affects the quality of their working life. During work, it creates a sense of flow that seems to benefit nurses’ mental well-being and avoid fatigue [17].

Thus, we are reminded that it appears to be essential to understand and assess these issues. Therefore, this study was designed to close the knowledge gap in this field, which could improve healthcare workers’ response and allow organizations to continue offering safe and effective care. Therefore, the purpose of this study is to assess nurses’ perceptions of the professional practice environment and its relation to organizational dehumanization and work passion.

## Purpose of the study

Assess nurses’ perceptions of the professional practice environment and its relation to organizational dehumanization and work passion.

## Research questions

1. What is the level of nurses’ perception of the professional practice environment?
2. What are the levels of organizational dehumanization and work passion among nurses?
3. What is the relationship between the professional practice environment, organizational dehumanization and passion for work?

## Method

### Research design

A descriptive correlational research design was used to achieve the purpose of the study.

### Setting

The study was conducted in critical care units and inpatient departments at the National Liver Institute in Shebin Elkom City/Menoufia Governorate, Egypt.

### Subjects

The current study was conducted with staff nurses working in the previously mentioned settings.

**The sampling technique** included a convenience sample of staff nurses (n = 274) from total population 1200 staff nurses who work in critical care units and inpatient departments with a response rate of 81%.

The sample size was determined by using the Solvin formula to assess the sample size of staff nurses [19].

$${\text{n}}\,{\text{ = }}\,{\text{N}}\,{\text{/}}\,{\text{1}}\,{\text{ + }}\,{\text{N}}\,{\left( {\text{e}} \right)^2}$$

$${\text{1200}}{\mkern 1mu} {\text{/}}{\mkern 1mu} \left( {{\text{1}}{\mkern 1mu} {\text{ + }}{\mkern 1mu} {\text{1200}}{\mkern 1mu} {\mkern 1mu} \times {\mkern 1mu} {\mkern 1mu} {{\left( {{\text{0}}{\text{.05}}} \right)}^{\text{2}}}} \right){\mkern 1mu} {\text{ = }}{\mkern 1mu} {\text{300}}{\mkern 1mu} \,{\text{staff}}{\mkern 1mu} {\text{nurses}}{\text{.}}$$

The sample was increased to 339 for attrition rate.

**The inclusion criteria were** staff nurses with more than one year of experience who were available at the time of the study and who agreed to participate in the study.

### Instruments for data collection

Three different instruments were used:

Personal characteristics of the staff nurses included age, education level, current work setting, gender, and years of experience.

### Instrument one

**Nursing professional practice environment questionnaire**. It was developed by Erickson [20] to assess nurses’ perceptions of the professional practice environment. It contains 39 items in 8 dimensions: leadership and autonomy in clinical practice (5 items), control over practice (5 items), communication about patients (3 items), teamwork (4 items), handling disagreement and conflict (9 items), staff relationships with physicians (2 items), internal work motivation (8 items), and cultural sensitivity (3 items). It uses a five-point Likert scale ranging from 1 to 5 as follows: 1 (strongly disagree), 2 (disagree), 3 (neutral), 4 (agree), and 5 (strongly agree).

#### Scoring system

The total score ranged from 39 to 195. The respondents’ total scores were classified into two levels: less than 65% (39–126) were unfavorable (negative), and ≥ 65% (127–195) were favorable (positive) to the professional practice environment [20].

### Instrument two

Organization dehumanization scale: This instrument was developed by Caesens et al. [21] to assess nurses’ perceptions of being dehumanized by their organization. This tool has 11 items. The tool uses a five-point Likert scale ranging from one (1) (strongly disagree) to five (5) (strongly agree).

#### Scoring system

The total score ranged from 11 to 55. The respondents’ total scores were classified into two levels: a score of less than or equal to 50% (11–26) suggested that the nurses were not being dehumanized, and a score of more than 50% (27–55) indicated that the nurses were being dehumanized [21].

### Instrument three

Work passion scale. It was developed by Vallerand et al. [22] to assess the level of work passion among nurses. This tool has 14 items in 2 dimensions: harmonious passion (7 items) and obsessive passion (7 items). The tool uses a five-point Likert scale ranging from one (1) (strongly disagree) to five (5) (strongly agree).

#### Scoring system

The total score ranged from 14 to 70. The respondents’ total scores were classified into two levels: less than 50% (14–34) was considered low level, and ≥ 50% (35–70) was considered high level of passion for work [22].

## The instruments’ validation

### Translation and back-translation

The instruments were translated from English to Arabic to ensure that they were comprehensible and culturally relevant for the participants. This translation process followed a standard translation and back-translation procedure:

1. **Initial Translation**: The tools were translated into Arabic by a qualified translator fluent in both English and Arabic and familiar with the cultural nuances of both languages.
2. **Back-Translation**: Different translators, who were not involved in the initial translation and were also fluent in both languages, independently translated the Arabic version back into English. This step helps to check for consistency and accuracy in the translation.
3. **Comparison and revision**: The original English version and the back-translated English version were compared. Any discrepancies were discussed and resolved by a panel of experts, including translators and researchers, to finalize the Arabic versions of the instruments.

### Reliability test for the study instruments

The Cronbach alpha reliability test in the current study yielded a reliability coefficient of 0.914 for the nursing professional practice environment questionnaire, 0.962 for the organization dehumanization scale, and 0.904 for the work passion scale.

### Pilot study

A pilot study was conducted to assess the feasibility and applicability of the questionnaires and determine the time needed for data collection. It was conducted on 10% of the nurses studied (33), who were included in the final analysis because no modifications occurred.

### Data collection procedure

Official written permission was obtained from the hospital administration, while oral consent from staff nurses. Once the clarity of the tools was confirmed, the study’s purpose and tools’ content were explained to each nurse who agreed to participate. They were asked to complete the tool and return it anonymously by the end of the day or at the latest, the next day. Data was gathered during morning and afternoon shifts, with nurses completing questionnaires in the presence of researchers to ensure all questions were addressed. It was made clear that participation in this study was voluntary, and participants could opt-out if they chose. Additionally, the researcher’s confidentiality and data protection measures were clearly articulated, ensuring full respect for participants’ privacy. Each staff nurse took 25–40 minutes to complete the questionnaires. The data collection stage of the study was applied for three months from 15/11/2023 to 14/2/2024.

### Ethical considerations

The study was conducted with careful attention to the ethical standards of research and the rights of the participants. The respondent’s rights were protected by ensuring voluntary participation; thus, oral informed consent was obtained after the purpose of the study was explained, the time of conducting the study, the potential benefits of the study, how the data would be collected, and the respondent’s right to withdraw from the research study at any time of violation of his/her rights.

The respondent was assured that the data would be treated as strictly confidential by coding it; furthermore, the respondent’s anonymity would be maintained as they would not mention their names, and the protocol of the study was revised and accepted by the Ethical and Research Committee (No. 975, date 21/6/2023) at the Faculty of Nursing, Menoufia University, before starting the study.

### Statistical analysis

Data cleaning and preparation were performed by checking for missing data and outliers and converting Likert scale responses into numerical values for analysis. Data analysis was performed via IBM SPSS Statistics for Windows, version 28 (IBM Corp., Armonk, N.Y., USA). The Shapiro-wilk test was used to show normal distribution of data. The reliability of the study tools was tested via Cronbach’s alpha value, which provides a measure of internal consistency. Descriptive statistical tests were used (frequencies, percentages, means, and standard deviation). One-way ANOVA tests also were used in this study for variables that had more than two groups such as educational level, age, and years of experience. Pearson correlation coefficients (r) were used to detect association between three variables. In addition, multiple linear regression analysis was undertaken to evaluate predictors of professional practice environment. All these tests were used as tests of significance at P < 0.05.

## Results

Table 1 shows that the highest percentage of the study sample was female (95.6%), and nearly half of them (47.8%) had ages ranging from 25 to less than 35 years old. Regarding educational level, they had an associate degree in nursing (42.34%). More than half of the sample worked in critical care units and had experience ranging from 5 to less than 10 years (56.2% and 56.57%, respectively).

**Table 1:** Socio-demographic characteristics of the study participants (n = 274)

Items	No.	%
**Age of nurses**		
15- <25 years	48	17.52%
25- <35 years	131	47.81%
35- <45 years	82	29.93%
45 and above	13	4.74%
**Nursing education level**		
Diploma in nursing	88	32.12%
Associate degree in nursing	116	42.34%
Bachelor in nursing	70	25.55%
**Workplace**		
Critical care units	154	56.20%
Inpatient departments	120	43.80%
**Gender**		
Male	12	4.38%
Female	262	95.62%
**Years of experience**		
< 5 years	60	21.90%
5- <10 y	155	56.57%
10- <15 y	40	14.6%
15 and above	19	6.93%

**Fig. 1:**
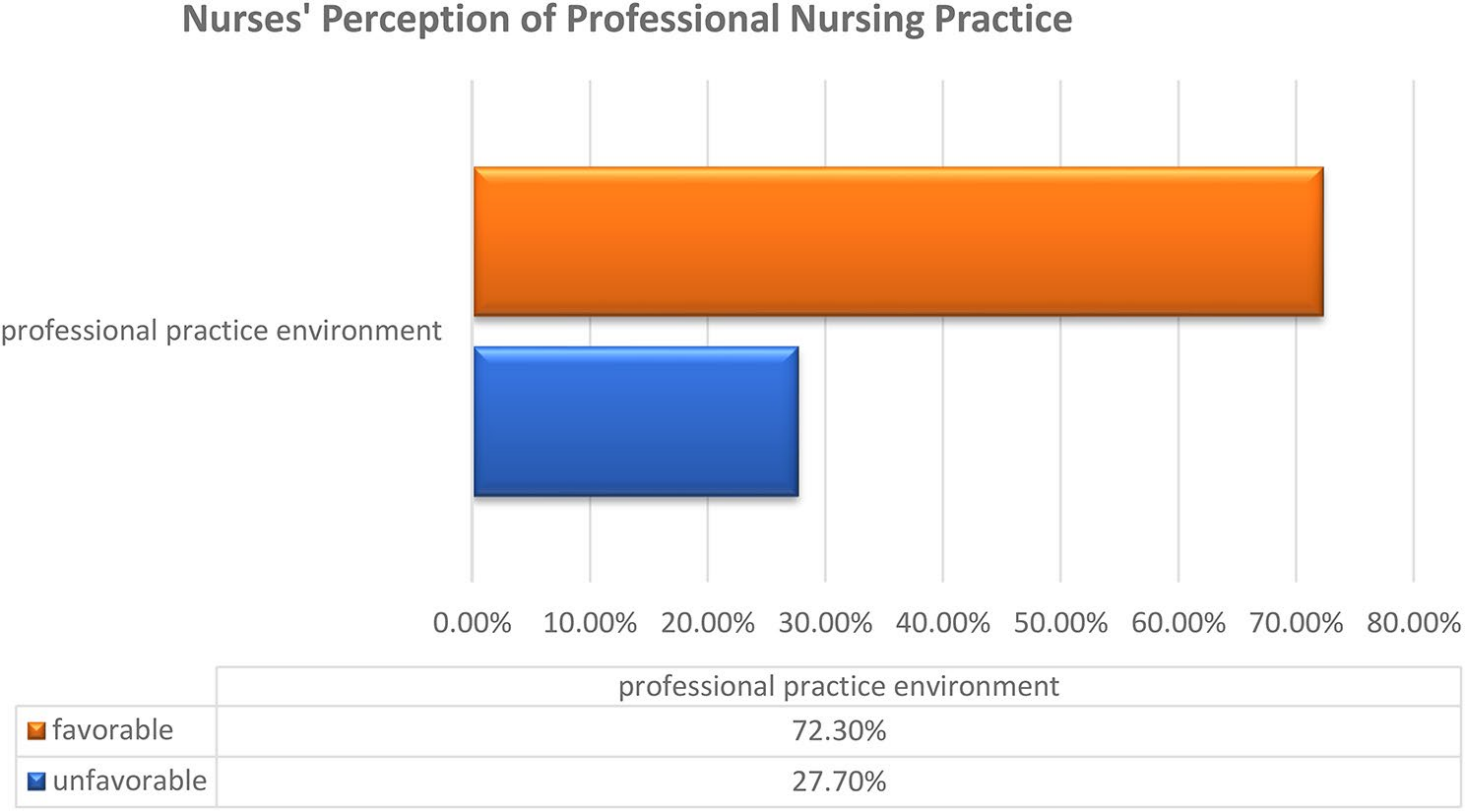
Percentage distribution of the levels of the professional practice environment as perceived by the staff nurses studied (n = 274)

Figure 1 represents that more than two-thirds of staff nurses studied (72.3%) had a favorable work environment. whereas more than one quarter (27.7%) of them had an unfavorable work environment.

**Fig. 2:**
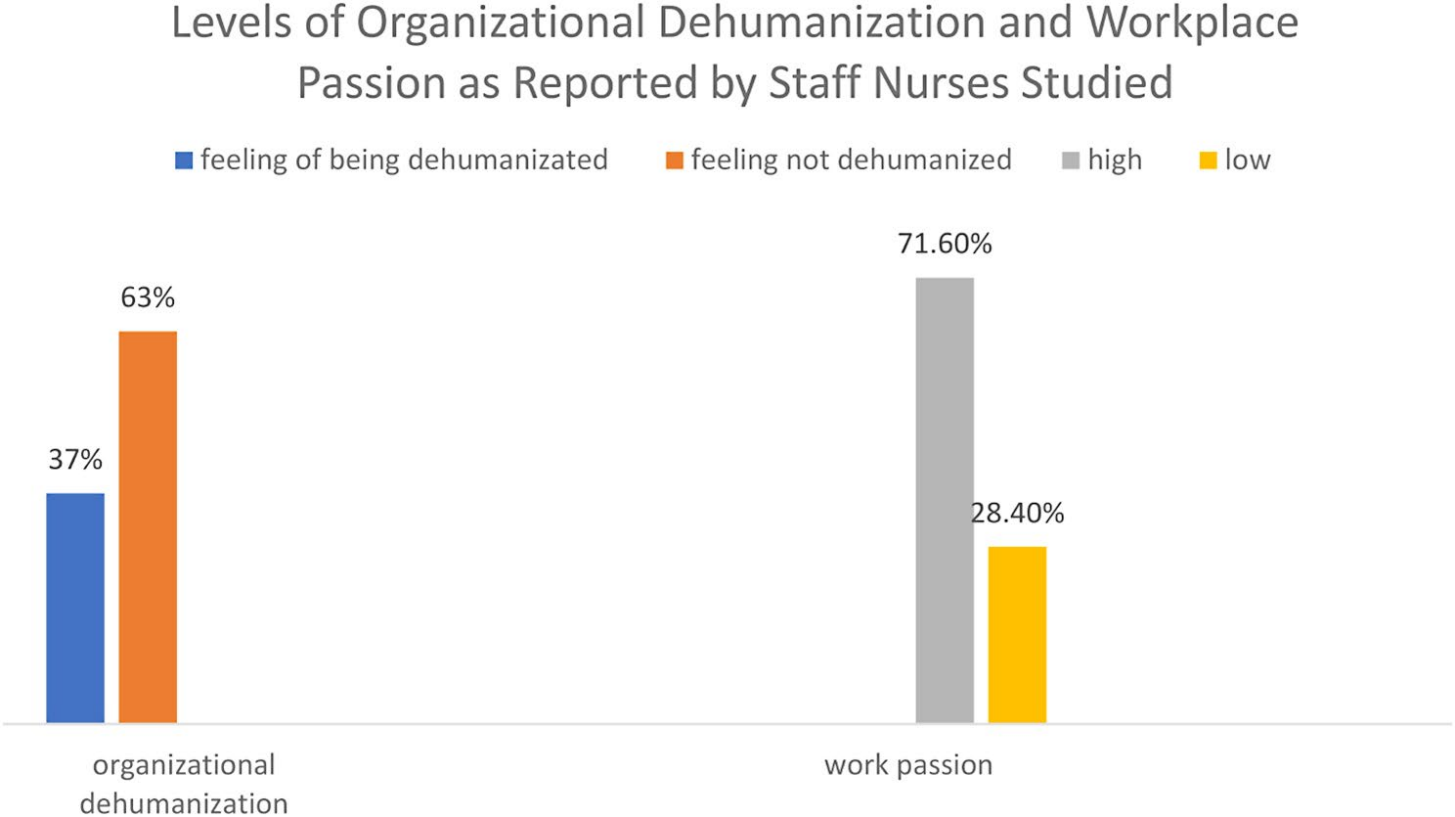
Level of organizational dehumanization and work passion as reported by the studied nurses (n = 274)

According to Fig. 2, more than two-thirds of nurses (71.6%) were highly passionate about their work. More than half of the nurses (63%) felt that their organization did not dehumanize them.

The overall mean score for all aspects of the professional practice environment as reported by staff nurses was 141.61 ± 13.89, as indicated in Table 2. The teamwork dimension had the highest mean percentage (83.63%) and the highest mean score (16.72 ± 2.66). The final ranking was associated with communication about the patient dimension, with the lowest mean percentage (63.75%) and mean score of 9.56 ± 1.41. The total mean score for workplace passion was 50.1 ± 7.25. The high score (72.28%) and first rank of work passion dimensions was harmonious with a mean score of 25.29 ± 4.16.

**Table 2:** Mean scores of the professional practice environment and workplace passion domains as reported by staff nurses (n = 274)

Domains	Min score	Max score	Mean ± SD	Mean %	Ranking
**Domains of professional practice environment**					
Leadership and autonomy in clinical practice	11	21	16.39 ± 1.44	65.56%	7
Control over practice	11	21	16.58 ± 1.81	66.35%	6
Communication about patient	5	13	9.56 ± 1.41	63.75%	8
Teamwork	10	20	16.72 ± 2.66	83.63%	1
Handling disagreement and conflict	20	43	32.2 ± 4.15	71.57%	5
Staff relationships with physicians	4	10	7.77 ± 1.17	77.77%	2
Internal work motivation	15	40	30.75 ± 7.22	76.88%	4
Culture sensitivity	8	15	11.6 ± 2.59	77.4%	3
**Total**	101	173	141.61 ± 13.89		
**Domains of work passion**					
Harmonious passion	14	35	25.29 ± 4.16	72.28%	1
Obsessive passion	17	35	24.80 ± 3.44	70.87%	2
**Total**	37	70	50.1 ± 7.25		

Table 3 demonstrates that, with the exception of workplace, there were significant differences in professional practice environment among nurses based on their personal characteristics: age, educational level, and years of experience (p = 0.003, 0.000, and 0.041, respectively).

**Table 3:** Mean differences among nurses regarding professional practice environment according to personal characteristics (n = 274)

Items	N	Mean	SD	F	p-value
**Age**					
15- <25 years	48	139.65	10.99	4.85	0.003
25- <35 years	131	144.6	13.56		
35- <45 years	82	137.66	15.08		
45 and above	13	143.69	12.94		
**Nursing education level:**					
Diploma in nursing	88	147.06	14.07	25.87	0.000
Associate degree in nursing	116	142.91	10.60		
Bachelor in nursing	70	132.63	14.31		
**Workplace**					
Critical care units	154	140.70	11.64	−1.18	0.219
Inpatient departments	120	142.78	16.32		
**Years of experience**					
< 5 years	60	140.45	12.60	2.79	0.041
5- <10 y	155	140.37	14.67		
10-<15y	40	145.13	11.45		
15 and above	19	148.00	13.99		

The mean difference is significant at the 0.05 level

Table 4 clarifies that significant differences in organizational dehumanization were found among nurses according to their personal characteristics; age, educational level, and workplace (p = 0.000, 0.000, and 0.022, respectively) except for years of experience.

**Table 4:** Mean differences among nurses regarding organizational dehumanization according to personal characteristics (n = 274)

Items	N	Mean	SD	F	p-value
**Age**					
15- <25 years	48	20.10	7.05	6.56	0.000
25- <35 years	131	18.02	7.83		
35- <45 years	82	23.77	12.58		
45 and above	13	23.15	9.94		
**Nursing education level:**					
Diploma in nursing	88	19.67	8.74	14.80	0.000
Associate degree in nursing	116	17.82	7.32		
Bachelor in nursing	70	25.40	12.46		
**Workplace**					
Critical care units	154	19.16	8.29	−2.21	0.022
Inpatient departments	120	21.88	11.26		
**Years of experience**					
< 5 years	60	19.57	7.22	0.78	0.508
5- <10 y	155	20.95	10.84		
10-<15y	40	18.70	8.87		
15 and above	19	21.42	9.55		

The mean difference is significant at the 0.05 level.

Table 5 illustrates that there was a significant difference in workplace passion among nurses with varying educational levels (F = 7.03, p = 0.001). While the rest of the personal characteristics, there was no significant differences in workplace passion among nurses according to age, department, and years of experience.

**Table 5:** Mean differences among nurses regarding workplace passion according to socio-demographic characteristics (n = 274)

Items	N	Mean	SD	F	p-value
**Age**					
15- <25 years	48	50.13	7.51	1.14	0.335
25- <35 years	131	50.85	7.42		
35- <45 years	82	49.07	6.85		
45 and above	13	48.92	6.96		
**Nursing education level:**					
Diploma in nursing	88	51.00	7.82	7.03	0.001
Associate degree in nursing	116	51.08	7.03		
Bachelor in nursing	70	47.36	6.21		
**Workplace**					
Critical care units	154	49.42	7.52	−1.80	0.076
Inpatient departments	120	50.98	6.83		
**Years of experience**					
< 5 years	60	49.67	7.76	0.369	1.05
5- <10 y	155	49.97	7.25		
10-<15y	40	51.85	6.93		
15 and above	19	48.84	6.16		

The mean difference is significant at the 0.05 level.

Table 6 illustrates that there was a positive, highly significant correlation between perception of professional practice environment and work passion (r =.793, p =.00). While there was a negative significant correlation between staff nurses’ perception of professional practice environment and organizational dehumanization (r =.467, p =.000). Additionally, there was a negative correlation between organizational dehumanization and work passion.

**Table 6:** Correlation between staff nurses’ perception of professional practice environment, organizational dehumanization and workplace passion (n = 274)

Variable		Organizational dehumanization	Workplace passion
**Professional practice environment**	**r**	−.467**	.793**
	**p-value**	**0.000**	**0.00**
**Organizational dehumanization**	**r**		**−.158**
	**p-value**		**0.01**

r: spearman correlation ** correlation is significant at the 0.01 level (2-tailed).

Table 7 presents a multiple linear regression model for factors affecting the professional work environment among the studied nurses. The model explained a significant portion of the variance in professional work environment (r^2^ = 0.75, p < 0.001). Additionally, passion (beta = 1.412) positively and significantly contributed to the perception of the professional work environment. Moreover, dehumanization (beta = − 0.498) negatively and significantly impacted the perception of the professional work environment. This highlighted the critical role of reducing dehumanization and fostering workplace passion to improve the professional work environment for nurses, as the following equation shows:

$$\begin{aligned} {\text{Professional work environment}} &= 80.9 + 1.412 \left( {{\text{work passion}}} \right) \\ & - 0.498 \left( {{\text{organizational dehumanization}}} \right). \\ \end{aligned} $$

**Table 7:** Multiple linear regression model for factors affecting professional work environment among the nurses studied (n = 274)

Model	Unstandardized coefficients		T	p-value
	Beta	Std. Error		
(constant)	80.976	3.25	24.88	0.00
Organizational Dehumanization	−0.498	0.04	−11.34	0.00
Workplace passion	1.412	0.06	23.88	0.00
R = 0.865, R^2\^ = 0.75, F = 402.57, P<0.001*				

F, p: F and p values for the model

R^2^: coefficient of determination

R: coefficient of regression

Beta: standardized coefficient

t: t-test of significance

## Discussion

The 2022–2030 Working for Health Action Plan reiterated the importance of investing in healthy, safe, and supportive practice environments for healthcare workers [23]. These environments not only improve health worker recruitment and retention but also contribute significantly to quality patient care and the overall strengthening of health systems [24, 25] (Al Sabei et al., 2021; Alzahrani, 2022). As a result, enhancing the work or practice environments for nurses at the hospital level has the potential to increase health system performance [26]. The current study aimed to assess nurses’ perceptions of the professional practice environment and its relation to organizational dehumanization and work passion among staff nurses who work in critical care units and inpatient departments at the National Liver Institute in Shebin Elkom city/Menoufia Governorate, Egypt.

The findings of the present study concerning the personal characteristics of staff nurses indicated that most of the participants were female. In terms of age, less than half of the sample was within the range of 25–34 years and held an associate degree in nursing. Furthermore, slightly over half of the nurses were employed in critical care units and had professional experience ranging from 5 to less than 10 years.

Regarding the professional practice environment, the study highlighted that a significant proportion of participants reported a favorable/positive professional practice environment. This environment plays a pivotal role in enabling healthcare providers to deliver high-quality patient care, as it contributes to reducing hospital-acquired infections, mortality rates, readmissions, and adverse events [2]. From the researchers’ viewpoint, this could be attributed to the accreditation of the National Liver Institute, which endows the institute with characteristics that foster a professional practice environment. The key attributes include involving nurses in decision-making processes regarding patient care and hospital policies, granting them autonomy in clinical practice, and promoting positive relationships with physicians, therapists, and other healthcare professionals. Additionally, the Liver Institute consistently invests in the growth and development of its staff, which often creates an environment where nurses feel motivated, appreciated, and well prepared to succeed.

This finding aligns with previous research exploring nurses’ perceptions of the professional practice environment. For example, studies by [27, 28] indicated that nurses generally expressed favorable or positive perceptions concerning the professional practice environment. Similarly, the research by Ibrahim et al. [29] revealed that participants exhibited a high level of perception regarding the professional practice environment.

In contrast, a study by Lucas et al. [30] revealed that nurses perceived their professional practice environment as mixed and generally unfavorable. Similarly, Lambrou et al. [31] reported that nurses had a moderate perception of their professional practice environment.

In an analysis of the dimensions within professional practice environments, the highest mean score and top-ranking dimension was teamwork, whereas the lowest mean score and last-ranking dimension was communication about patients. Researchers attribute the high score for teamwork to the presence of healthy, respectful relationships among nurses, their colleagues, and other healthcare providers, which fosters a sense of unity and team spirit. Conversely, the lowest mean score in communication about patients is linked to limited information sharing, incomplete patient details, inadequate handover practices, and excessive workload, all of which contribute to its lower ranking.

In line with the findings of the current study, Rivaz et al. [32] highlighted that among the dimensions of the professional practice environment, teamwork held the top rank, whereas communication regarding patient and staff relationships with physicians ranked the lowest. In a related study, Zeleníková et al. [18] reported that “leadership and autonomy in clinical practice,” alongside “teamwork”, were the highest-ranked aspects within the professional practice environment, whereas communication about patients occupied the lowest position.

Contrary to the findings reported by Lorenz and Guirardello [33], who identified “control over the practice environment” as the dimension with the highest score in the professional practice environment and “autonomy” as the lowest score, Ibrahim et al. [29] presented differing results. Their study indicated that the highest mean score in the professional nursing practice environment pertained to collegial nurse‒physician relationships, whereas the lowest mean score was associated with the adequacy of staffing and resources.

In examining organizational dehumanization, the current study revealed that over two-thirds of the nurses surveyed did not feel dehumanized by their organization. This outcome can likely be attributed to the presence of a supportive professional practice environment that fosters team cohesion, autonomy, and effective leadership. These findings are aligned with research by Aly et al. [34] (2023), who similarly reported that a majority of participants experienced minimal levels of dehumanization.

Abou Zeid et al. [8] disagreed with this study, claiming that organizational dehumanization was moderate. According to the findings of the study by Hashish et al. [35], only forty percent of the nurses thought that the organization dehumanized them to a considerable degree. Additionally, Bashandy et al. [36] reported that more than half of nurses perceived organizational dehumanization at a moderate level.

In terms of workplace passion, the present study revealed that staff nurses were very passionate about what they did. This outcome can be attributed to various factors, including the recognition and support they receive from their managers, the autonomy they exercise in their roles, and the strong sense of camaraderie and teamwork prevalent within their workplace environment.

In the same context, a study by Aly et al. [34] revealed that the majority of nurses demonstrated a strong passion for their work. Similarly, Villar et al. [37] reported that the nurses under the study presented high levels of work-related passion, attributing this outcome to a supportive nurse manager and a positive work environment. Additionally, Zito et al. [13] reported that staff nurses were highly passionate about their employment.

The present study identified a statistically significant negative correlation between the professional practice environment and organizational dehumanization. This finding suggests that features of the professional setting at the Liver Institute, such as nurses exercising greater autonomy, fostering collaborative teamwork between nurses and doctors built on mutual respect and trust, and the inclusion of staff nurses in decision-making processes, contribute to mitigating organizational dehumanization. According to Ahmed and Mohamed’s research [38], nurses’ perceptions of organizational dehumanization diminish and a more favorable work environment results when they experience fairness and support from their managers and supervisors.

Statistical analysis revealed a statistically significant positive association between nurses’ job passion and their professional practice environment. This outcome can be attributed to several pivotal factors within the workplace. Nurses who perceive their practice environment as supportive and empowering—characterized by recognition of their contributions, managerial support, and opportunities for active participation in decision-making—experience heightened levels of enthusiasm and commitment to their work. Furthermore, the collaborative dynamics among nurses, as well as other members of the healthcare team, foster a strong sense of teamwork and mutual respect. Collectively, these elements contribute to enhancing the nurses’ professional passion and engagement.

The findings of the current study align with those of Peyton and Zigarmi [14], who identified a statistically significant positive correlation between the professional practice environment and passion work within their sample. Similarly, Borchardt [39] highlighted that an organization’s professional practice environment plays a pivotal role in influencing nurses’ work passion, as well as their recruitment and retention.

The present study found a statistically significant negative correlation between organizational dehumanization and passion for work. From the perspective of the researchers, this result can be attributed to the presence of a motivating work climate, a healthy work environment, and supportive supervisors among staff nurses. These factors fostered elevated levels of work passion, which, in turn, diminished feelings of dehumanization in their professional roles. The findings of the present study align with those of Aly et al. [34], who similarly reported a statistically significant inverse relationship between workplace passion and organizational dehumanization.

## Limitations

The study utilized a self-administered questionnaire, which may lead to potential biases. Additionally, the study concentrated on nurses from a particular region, which could restrict the broader applicability of its findings. Future research utilizing objective measures and encompassing the various regions and healthcare settings is suggested to improve both accuracy and reliability and enables the broader application of findings. Moreover, researchers have identified a lack of sufficient studies exploring and clarifying the relationship between the professional practice environment and organizational dehumanization. Furthermore, there is a scarcity of research examining the connection between organizational dehumanization and work passion.

## Conclusion

This study explored nurses’ perceptions of the professional practice environment and its relation to organizational dehumanization and work passion. The findings indicated a statistically significant negative relationship between the professional practice environment and organizational dehumanization. Conversely, workplace passion was found to have a positive and significant impact on perceptions of the professional practice environment. The results underscore the importance of minimizing dehumanization and promoting workplace passion to enhance the professional work setting. The professional practice environment plays a crucial role in nurturing nurses’ passion for their work by fostering a sense of value, respect, and empowerment to deliver high-quality care. A positive and supportive workplace also encourages autonomy in decision-making and strengthens interdisciplinary collaboration and communication. These findings offer essential theoretical and practical insights for future research and initiatives aimed at enhancing nursing practice environments—an integral component in improving the quality of patient care.

## Implication for practice

The results of this study suggest that the establishment of a favorable practice environment is of importance in guaranteeing to lessen feelings of being dehumanized and increase nurses’ levels of work passion. So, a better understanding of how nurses’ work environment and passion for their work relate to their intentions to perform well for their organization is needed.

Additionally, dehumanization is an undesirable concept that lessens originality. As a result, people may decide to quit supporting an organization. As a result, managers must design interventions to teach staff members that they are valued as individuals rather than expendable goods. To make staff members feel less dehumanized and more supported, hospitals and their managers may apply particular human resources practices, such as lowering workload, enhancing job stability, and providing training and development opportunities for their growth and grooming. Workshops, conferences, and team-building exercises involving active engagement between individuals from various levels of management are far better at reducing employees’ sense of dehumanization. Organizations must understand that treating employees as human beings comes before considering their performance.

Moreover, organizations should encourage and facilitate research on work passion, as passion can lead to optimal experiences that protect against a sense of dehumanization. Also, organizations should encourage their staff to adopt a harmonious passion, as this may lead them to experience flow more often and consequently experience fewer burnout symptoms than in work environments that facilitate the adoption of an obsessive passion [13]. Additionally, further research is needed to explore the relationship between the professional practice environment and organizational dehumanization. Furthermore, it is recommended that future research explore the effects of personal characteristics—such as marital status, religious beliefs, and family income—on the perception of the professional practice environment among nurses.

## Data Availability

The corresponding author can provide the data utilized upon request.
